# Editors’ Note

**DOI:** 10.63144/ijt.2026.6766

**Published:** 2026-06-01

**Authors:** Ellen R. Cohn, Jana Cason

## Submissions for the Next Issue

The International Journal of Telerehabilitation (IJT) will accept new submissions August 1, 2026 – September 10, 2026.

## Welcome to Senior Associate Editor Kyrylo Malakhov

We are delighted that researcher Kyrylo Malakhov has joined IJT as Senior Associate Editor. Kyrylo has served as an IJT author, reviewer, editorial advisor, and guest editor. He is currently completing his doctoral dissertation and is a Research Fellow in Computer Science, Microprocessor Technology Lab/Department, V.M. Glushkov Institute of Cybernetics of the National Academy of Sciences of Ukraine. Kyrylo earned an MSc degree in Computer Science, and a Bachelor of Philology in English and Foreign Literature at Luhansk Taras Shevchenko National University. Since 2023, Kyrylo has been a member of the Interdepartmental Working Group on the Development of Telemedicine Implementation in Ukraine. Since 2024, he is a member of the Coordination Scientific Council of the National Academy of Sciences of Ukraine on Artificial Intelligence. Selected links: https://linktr.ee/malakhovks

## Journal Overview

IJT is a biannual journal dedicated to advancing telerehabilitation by disseminating peer-reviewed information about current research and practices. IJT is indexed by PubMed and Scopus. IJT is published via Open Journal Systems (OJS) and is sponsored by Hawaiʻi Pacific University. This institutional support enables IJT to be subscription-free and require no author fees.

IJT enjoys a diverse international audience due to the expanding global relevance of telemedicine, telehealth, and telerehabilitation. In the current issue we are pleased to feature the work of authors from: Australia, Canada, Germany, India, Italy, Jordan, Nigeria, Turkey, Ukraine, United Arab Emirates, and the United States of America. In the spirit of intellectual collaboration across borders, it is heartening that articles are increasingly co-authored by researchers from different countries and often multiple institutions.

We are grateful to our diligent and generous peer reviewers who donate their time and hail from multiple disciplines. Their suggestions invariably elevate the work of even our most experienced authors. Our reviewers are not named herein, to preserve the anonymity of their reviews.

Sincerely,

Jana Cason, DHSc, OTR/L, FNAP, FATA, FAOTA Ellen R. Cohn, PhD, CCC-SLP, ASHA-F, FATA, NAP-F IJT Co-Editors-in-Chief

